# Compound Odontoma Associated with Dentigerous Cyst Incidentally Detected in an Adult Patient: Tomography and Histological Features

**DOI:** 10.1155/2022/6210289

**Published:** 2022-05-02

**Authors:** Mattia Manfredini, Susanna Ferrario, Luca Creminelli, Elisabetta Kuhn, Pier Paolo Poli

**Affiliations:** ^1^Implant Center for Edentulism and Jawbone Atrophies, Maxillofacial Surgery and Odontostomatology Unit, Fondazione IRCCS Cà Granda Ospedale Maggiore Policlinico, Milan, Italy; ^2^Department of Biomedical, Surgical and Dental Sciences, University of Milan, Milan, Italy; ^3^Division of Pathology, Fondazione IRCCS Ca' Granda Ospedale Maggiore Policlinico, 20122 Milan, Italy

## Abstract

Odontoma is the most common benign odontogenic tumor of epithelial and mesenchymal origin. The standard treatment involves a conservative approach. While this procedure is generally well accepted and tolerated, some difficulties may arise in case of odontomas associated with cystic lesions. In general, the expansive nature of cystic lesions requires their surgical excision, different from isolated nonsymptomatic odontomas that can be monitored radiographically. However, to the best of our knowledge, there is scarce evidence currently available reporting on the presence of odontoma-associated cystic lesions in the oral cavity. Therefore, the present case report is aimed at describing the diagnostic clinical, radiological, and histological features together with the surgical management of a dentigerous cyst associated with a compound odontoma. Following surgical removal of the lesion, no recurrence was observed after 12 months of follow-up.

## 1. Introduction

Odontomas are benign proliferations of odontogenic origin, characterized by a mixed histopathological composition, being formed by epithelial and mesenchymal cells with dental hard tissues [1–5]. They are considered hamartomatous malformations producing mineralized dental tissues, rather than true benign neoplasms. Although they are the most frequently detected benign tumors in the clinical practice, their aetiology remains unclear. Typical clinical signs include retention of deciduous teeth, delayed eruption, presence of inclusions, and increased gingival volume among others. Odontomas are most commonly observed in patients during their second decade of life, with no apparent gender susceptibility, being equally distributed in males and females [6, 7].

From a histomorphological standpoint, odontomas are divided into two different entities according to the latest classification of the World Health Organization [8]: compound and complex. The former is characterized by small variable formations structurally similar to teeth, while the complex form has no anatomical similarity to teeth and appears as an undefined mass of enamel and dentin [9].

Odontomas can be observed in both the jaws, are generally symptomless, and are typically discovered accidentally following routine radiological examinations performed for other oral conditions. Radiographically, they appear as radiopaque lesions, consisting of a cluster of more or less developed tooth structures. A radiolucent band is often visible around the radiopaque mass. In this context, odontomas and osteomas can be hardly distinguishable radiographically. The calcified component has a mixture of enamel, tubular or dysplastic dentine, epithelial-connective matrix producing enamel, and connective tissue [3].

If the diagnosis of odontoma is certain, it is generally not necessary to remove the lesion, unless it prevents tooth eruption or gives rise to complications, such as the development of cystic lesions. In these cases, surgical removal might be indicated.

Both odontomas and odontogenic cysts are frequently encountered in dental practice; however, the simultaneous presence of both lesions in the same anatomical region is somewhat rare. In addition, the diagnostic hypothesis is solely based on the radiographic appearance. The interpretation is therefore uncertain, as these lesions can be misdiagnosed for other radiographically similar fibro-osseous lesions, such as fibrous dysplasia and fibroma ossificans [10]. In this respect, there is still little evidence concerning the diagnostic workup of combined cystic and tumor lesions. Thus, the purpose of the present report was to describe the diagnostic and surgical aspects in a case of a compound odontoma associated with a dentigerous cyst in the mandibular interforaminal region.

## 2. Case Presentation

The present clinical case is reported in accordance with the CARE guidelines (http://www.care-statement.org) for improving the quality of reporting of case reports. All treatment procedures were performed in compliance with the Declaration of Helsinki on ethical principles for medical research involving human subjects.

A 65-year-old white Caucasian woman came to the authors' attention sent by her private dentist seeking for a consultation to evaluate the removal of a lesion located in the interforaminal region of the mandible. The medical history was noncontributory.

**Figure 1:**
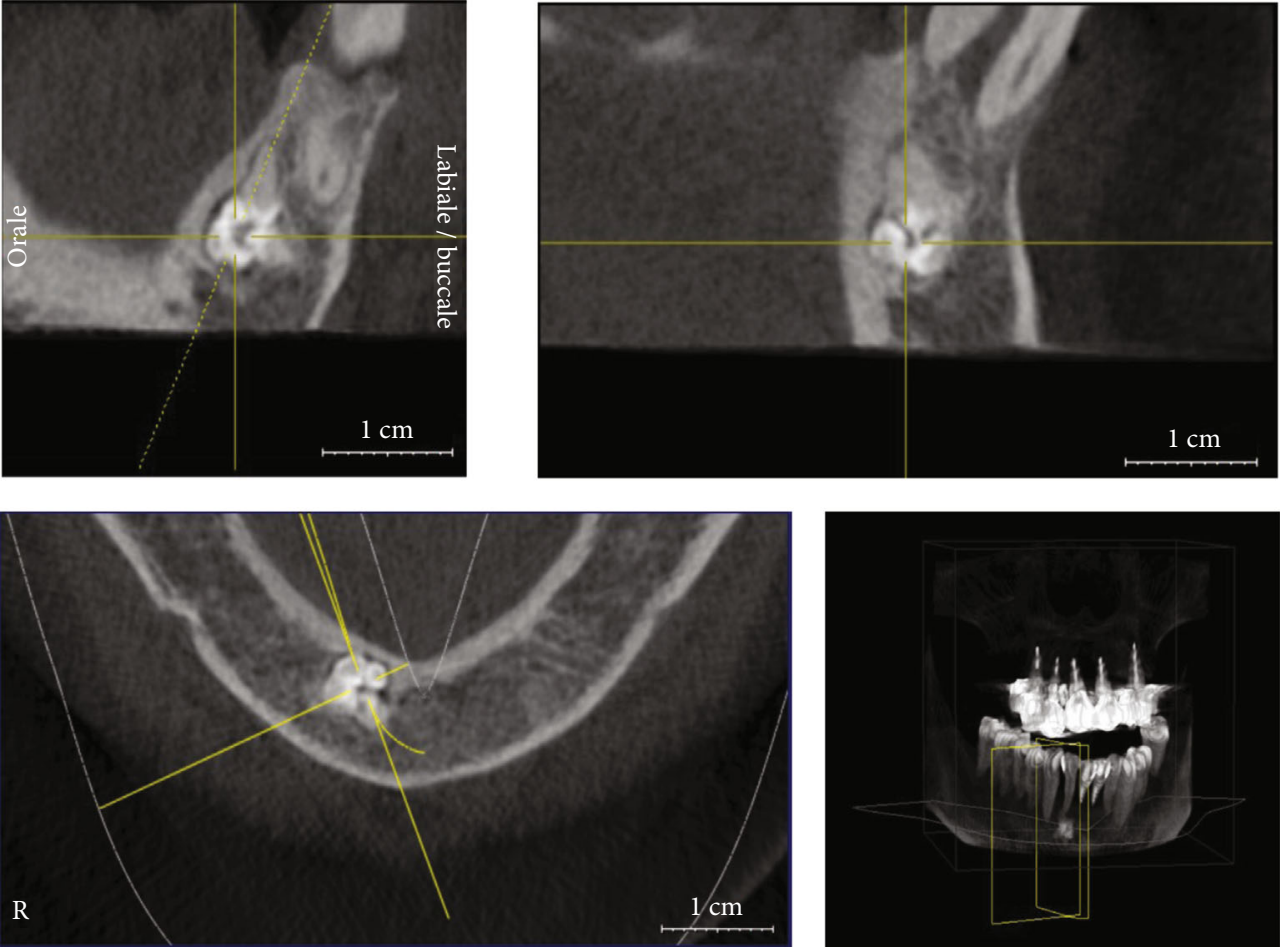
The image shows the size of the lesion in the CBCT scan approximately 10 years before its removal.

**Figure 2:**
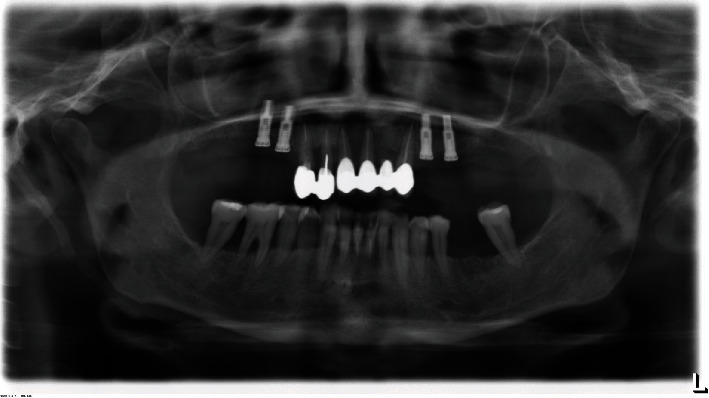
Orthopantomography performed in 2013.

**Figure 3:**
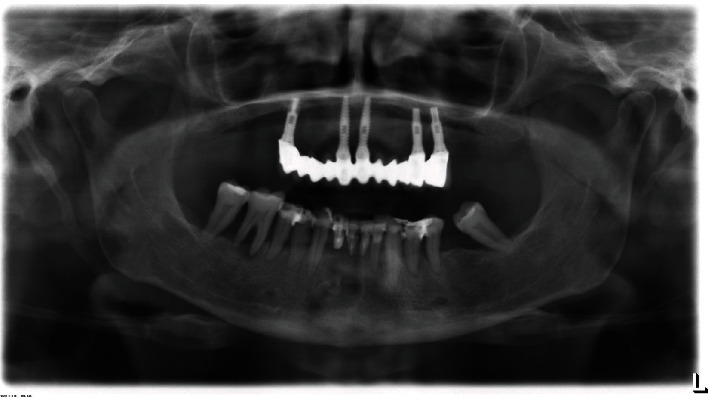
Orthopantomography performed in 2019.

**Figure 4:**
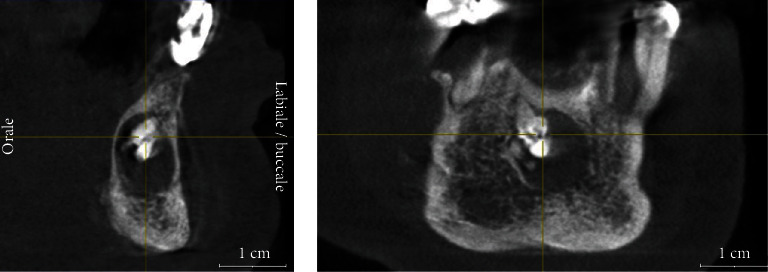
CBCT scan performed in 2020, showing the enlargement of the lesion compared to the first CBCT scan.

The patient underwent an initial cone beam computed tomography (CBCT) scan in 2010 and two different orthopantomographs, in 2013 and 2019, respectively (Figures 1–3). The examination of the patient's radiological records revealed an osteolytic cystic-like lesion associated with a radiopaque solid mass delimited by radiopaque margins, involving the apices of the lower incisors. By comparing the two orthopantomographs, it was possible to appreciate an expansive growth of the lesion; therefore, a new CBCT scan was prescribed in 2020 (Figure 4). From the comparison of the multiplanar reconstructions obtained in the two CBCT scans, it was possible to confirm the enlargement of the lesion throughout the years. The expansive behavior of the lesion and the need for a histopathological analysis to confirm the final diagnosis led to the decision of a surgical approach.

Upon clinical examination, the interforaminal region showed no signs or symptoms of swelling, redness, or inflammation despite the patient's poor oral hygiene conditions. The lower incisors presented with no mobility, although they were splinted with composite resin to the neighboring teeth. The patient was asymptomatic.

**Figure 5:**
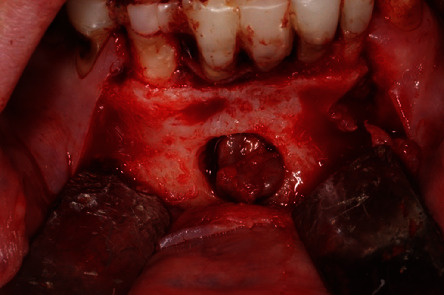
Vision of the vestibular bone hatch.

**Figure 6:**
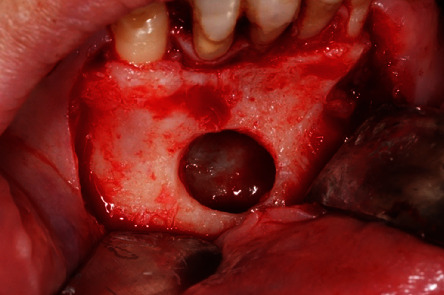
Removal of the lesion through the bone hatch.

**Figure 7:**
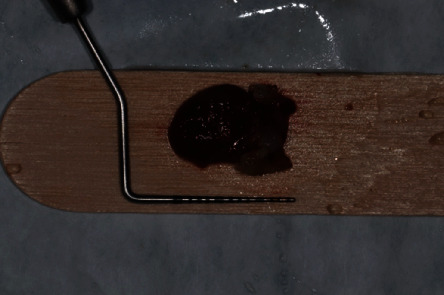
Lesion with millimeter probe.

The surgical procedure was performed on an outpatient basis under local anesthesia induced with mepivacaine hydrochloride 20 mg/mL with epinephrine 1 : 100.000 (Optocain, Molteni Dental Srl, Milan, Italy) injections. Bacterial decontamination was achieved with an antiseptic rinsing solution consisting of 0.2% chlorhexidine digluconate for 1 minute. To access the lesion, an intrasulcular incision was made with divergent releasing vertical incisions distally with respect to the right canine and the left lateral incisor. After flap elevation and soft tissue debridement, a thinned and more transparent buccal cortical wall was appreciated in correspondence with the lesion. A circular osteotomy was made with a surgical handpiece under copious irrigation with sterile saline to create a bony window and expose the lesion. At this point, a careful isolation of the encapsulated lesion and complete removal were accomplished with Lucas-like surgical curettes of different sizes and shapes. The entire lesion was excised in toto with the help of mosquito surgical forceps. The surgical defect was debrided to remove any soft tissue remnants and expose macroscopically healthy bleeding bone tissue. The surgical site was irrigated vigorously with sterile saline, and the flap was finally repositioned and sutured with 4/0 silk single stitches. The lesion measuring 15 × 10 × 7 mm was sent to the anatomical pathology department for histopathological examination (Figures 5–7).

**Figure 8:**
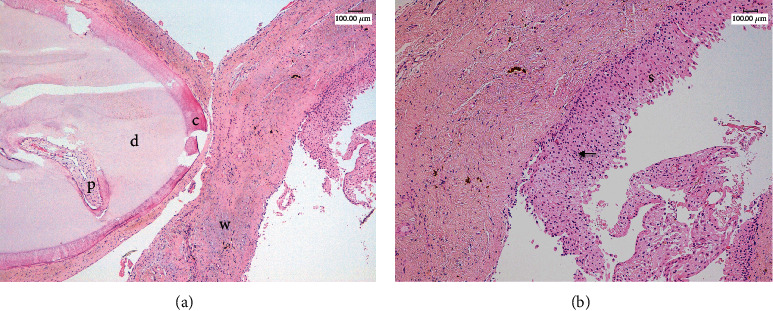
Representative histological pictures of the lesion. (a) The odontoma has a well-organized tooth-like structure composed mainly of dentin (d) with a thin outer layer of cement (c) and little inner dental pulp-like tissue (p). (b) It is appreciable a thin fibrous wall (w) with brown hemosiderin deposits of a cystic lesion ((a) 50x magnification). At higher magnification, the cyst is covered by a hyperplastic nonkeratinizing squamous epithelium (s) with exocytosis of neutrophils (arrow, (b) 100x magnification) (haematoxylin-eosin staining).

**Figure 9:**
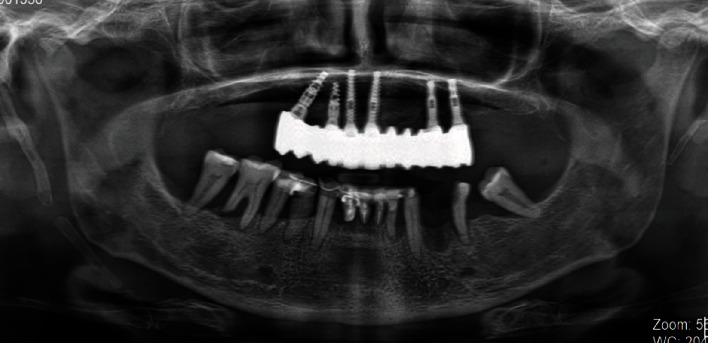
Orthopantomography performed in 2021.

From an initial macroscopic evaluation, the resected specimen consisted of a cystic lesion with a thin 1 mm diameter regular wall, associated to a tooth-like structure measuring 7 mm in its long axis. Histologically, the cyst had a thin fibrous wall with brown hemosiderin deposits, covered by a hyperplastic nonkeratinizing squamous epithelium, consistent with a dentigerous cyst. The tooth-like structure was mainly composed of dentin with a thin outer layer of cement and little inner dental pulp-like tissue, compatible with compound odontoma (Figure 8). No recurrence was observed at the 12-month follow-up recall (Figure 9).

## 3. Discussion

The present report described the surgical management of a compound odontoma associated with a cystic lesion in the interforaminal area. Heterogeneity can be found in literature regarding the epidemiology of odontomas. In this matter, our findings are inconsistent with other studies reporting that compound odontomas are more frequently diagnosed in patients during the first two decades of life [11] in the premaxillary region [12]. Furthermore, also the clinical and radiological features of the odontoma diagnosed herein do not resemble those commonly encountered for this type of lesion, being in intimate contact with an odontogenic cyst. Nonetheless, the radiographic evidence of the lesion allowed surgical intervention in good time, avoiding further complications and pathological expansion toward healthy structures.

Odontomas are odontogenic in nature, as they derive from the epithelial and/or mesenchymal tissues from which the teeth originate, and can induce cystic proliferation [13]. This cystic lesion results from degeneration of the enamel organ after partial or total development of the crown within the odontoma itself and arises following accumulation of fluid between reduced enamel epithelium and crown of an unerupted tooth [14]. Although these changes are mentioned as possible, they have been seldomly observed in the clinical practice. Dentigerous cyst is developmental in origin, mostly associated with an unerupted tooth or rarely with an odontoma. When they occur concurrently, it is possible to observe a combined lesion, increased in size, with the potential to expand towards noble structures [15].

Clinically, odontomas can present as intraosseous or extraosseous lesions. Intraosseous odontomas develop within mandibular or maxillary bone structures and, in some cases, may also erupt into the oral cavity. Peripheral ones arise at the level of extraosseous soft tissues, are very rare, and tend to exfoliate [16]. In the present case, the lesion was classified as intraosseous, being limited to the cortical plates of the symphysis region.

Typically, treatment of odontomas involves surgical removal, while therapeutic strategies reported for dentigerous cysts range from marsupialization to enucleation. This complies favorably with the therapeutic plan adopted in the present report, where surgical removal of the lesion was considered as the treatment of choice. Conversely, marsupialization is more commonly used in children when compared to enucleation because it is surgically less invasive and preserves the eruption of permanent teeth. Other factors favoring marsupialization include young age, lack of patient compliance for more invasive surgical treatment, tooth position and development, and eruptive potential [17, 18].

In the present case, the bidimensional radiographic features of the lesion were initially inconclusive for diagnostic purposes. Additional radiographic evaluation with CBCT scan, at a relatively low radiation dose and high resolution, was necessary to determine the real extent and characteristics of the lesion. Indeed, the bidimensional limitations of periapical and panoramic images did not allow a complete three-dimensional visualization of the anatomical region. In this regard, CBCT images are necessary not only to assess the lesion itself but also to identify possible associated pathologies, the proximity to noble structures, and to formulate a correct treatment plan [19–21].

The imaging features of fibro-osseous lesions are very difficult to be distinguished among the large group of similar pathologies such as fibroma ossificans and fibrous dysplasia.

In those cases, CBCT is an important tool for the differential diagnosis of such lesions [22]. In the present report, the CBCT images provided a better representation of the structures involved. In addition to the hyperdense image detected in the interforaminal region, a second area with a cystic appearance was noticed. These radiological features were critical during the therapeutic approach, allowing an ideal planning of the surgical procedure and clarification of the diagnosis. It should be noted that CBCT represents the gold standard radiological exam to exclude any suspect of inferior alveolar nerve involvement or to reveal any neurological involvement of the lesion [19, 20]. Nevertheless, histopathological analysis remains mandatory to confirm the initial diagnostic hypothesis. It is worth noting that ameloblastic odontomas and fibro-ameloblastic odontomas are rare but clinically and radiographically similar to common odontomas [23]. This strengthens the essential role of the histopathological analysis in the differential diagnosis of these lesions.

In the present report, the odontoma was associated with another lesion with cystic features. In this regard, a dentigerous cyst could have been probably the result of cystic degeneration and had to be chosen as the diagnostic hypothesis. This was confirmed after surgical removal of the lesion, as the histopathological analysis showed a compound odontoma associated with a dentigerous cyst. Despite their benign nature, these lesions are well recognized by their growth pattern and should be completely removed to avoid secondary complications and possible sequelae for the patient, with an excellent prognosis following surgery. As a matter of fact, no recurrence was observed after 12 months of follow-up.
